# Employment for Epileptics

**Published:** 1886-11-20

**Authors:** Nina Paget

**Affiliations:** 28, Boltons, S.W.


### EMPLOYMENT FOR EPILEPTICS.

Some of the "difficult cases" recorded in The Hospital show that the cases of epilepsy cause much widespread anxiety and trouble. I have taken much interest in the employment of epileptics, and am anxious to bring their wants before the public. I feel that if you could be induced to investigate the practicability of a plan which was laid before the Council of the Charity Organisation Society, and discussed by several of the most able and representative medical men holding high positions in the profession, it would confer a boon on one of the saddest class of sufferers. I should be happy to place at your disposal materials for articles; or I have a narrative written, showing the needs of these cases, which I should be happy to send you. Hoping you may feel it in your power to help a class who, though ill, are still willing to work,

28, Boltons, S.W. Nina Paget.

*** If Miss Paget will kindly forward the narrative and other papers, as she proposes, they shall have careful consideration.—Ed. T. H.

